# The Weather

**Published:** 1846-08

**Authors:** 


### THE WEATHER—THE HEALTH OF LOUISVILLE.

Reports from all quarters speak of intensely hot weather. We hear of 100°, and upwards, in Boston. In St. Louis on the 5th of July the thermometer is said to have risen in the shade to 102°; and for three days afterwards it ranged there from 96° to 99°. Some of the thermometers in this city indicated a temperature of 96° about the same time, but our own never stood higher than 93°. Sunday morning, July 12th, it stood at 80°, at sun rise, and, at 3 o'clock, at 92°. On the 15th it was down to 60° in the morning, and on the 16th as low as 56°—a range of 40° in four days, taking the highest figures named as correct. Our London Correspondent mentions that the same extreme heat has been experienced there, which is very unusual for that climate. Writing, June 17th, he says:

"You speak in your last letter of the temperature of Kentucky, and add, that I am not suffering in London from any such heat. You are mistaken; I do not remember ever having, in June, been so much affected by heat. That you may see what reason I have for complaining, and have what, as a meteorologist, I know will will interest you, I send you a table of the weather kept here during one week.

| | | Lowest previous night. | Highest in the shade. | Highest in the sun. | Average of extreme cold. |
|---|---|---|---|---|---|
| June 7 | - | 64 | 88 | 108 | |
| 8 | - | 62 | 79 | 98 | |
| 9 | - | 57 | 77 | 93 | |
| 10 | - | 57 | 70½ | 78 | 60·02° |
| 11 | - | 51 | 80 | 95 | |
| 12 | - | 62 | 83 | 101 | |
| 13 | - | 63 | 85½ | 110 | |
| 14 | - | 63 | 86 | 109 | |

"The average temperature by the shade thermometer was 81·92, and by the sun register 102·30; so that the first thirteen days of June may be said to have afforded a continuous average of temperature of about 82°."

The health of Louisville has continued good up to this time. The sexton reported the number of interments for the two weeks ending July 14th, to be adults, 15; infants, 18. Perhaps bowel complaints have prevailed more generally among children than for some summers past, but the mortality among that class of our population has not been great.

